# Living donor-gifted allograft lithiasis: surgical experience after bench surgery stone removal and follow-up

**DOI:** 10.1007/s00240-023-01463-1

**Published:** 2023-06-26

**Authors:** Alba Sierra, Carlos Castillo, Enric Carbonell, Maria Muní, Carmen Martinez, Juan Manuel López, Lluís Peri, Antonio Alcaraz, Maria Pilar Luque, Mireia Musquera

**Affiliations:** 1 Division of Kidney Transplant, Department of Urology, Hospital Clinic, Barcelona, Spain; 2 Departament de Cirurgia i Especialitats Medicoquirúrgiques, Facultat de Medicina i Ciències de la Salut, Universitat de Barcelona, Barcelona, Spain; 3 Division of Transplant Surgery, Department of General Surgery, Hospital General Plaza de la Salud, Santo Domingo, Dominican Republic

**Keywords:** Kidney transplant, Living donor, Urolithiasis, Flexible ureterorenoscopy (f-URS), Bench surgery

## Abstract

This study presents the surgical experience and long-term outcomes of living donor kidney transplantations involving asymptomatic kidney stones, using ex vivo flexible ureterorenoscopy (f-URS) during bench surgery for stone removal. Out of 1743 living kidney donors assessed between January 2012 and October 2022, 18 (1%) were diagnosed with urolithiasis. Among them, 12 donors were rejected, and 6 were accepted for kidney donation. Stone removal was successfully performed using f-URS during bench surgery, with no immediate complications or acute rejections observed. The study analyzed six living kidney transplants, of which 4 (67%) donors and three recipients were female, and 4 (67%) donors were blood-related to the recipient. The median age for donors and recipients was 57.5 and 51.5 years, respectively. The stones, primarily located in the lower calyx, had a median size of 6 mm. The median cold ischemia time during surgery was 41.6 min, and ex vivo f-URS ensured complete stone removal in all cases. After a median follow-up of 120 months, the remaining grafts were functioning well, and no urinary stone recurrence was observed in either the recipients or living donors. The findings suggest that bench f-URS is a safe approach for managing urinary stones in kidney grafts, providing good functional outcomes without stone recurrence in selected cases.

## Introduction

It has been widely demonstrated that early kidney transplantation is the best option for patients with end-stage renal disease, improving survival and quality of life compared with dialysis [1]. The scarcity of organs for transplantation has pushed professionals to find new strategies to increase organ availability, including living donation, donation after death determined by circulatory criteria (DCD), and the use of organs from expanded-criteria donors (ECD) and non-standard risk donors [2]

Nephrolithiasis is considered a relative contraindication for kidney donation due to the risk of adverse stone-related events in recipients [3]. Unfortunately, the incidence of nephrolithiasis has increased dramatically in the last 30 years and its diagnosis is gradually occurring earlier, probably due to environmental changes such as dietary habits [4]. To increase the number of valid grafts with appropriate management, several reports have demonstrated successful outcomes using kidney grafts with urolithiasis [5, 6]. Along that line, the Amsterdam Forum on the Care of the Live Kidney Donor outlined certain acceptance criteria for an asymptomatic potential donor with a history of a single stone, including (1) no hypercalciuria, hyperuricaemia, or metabolic acidosis; (2) no cystinuria or hyperoxaluria; (3) no urinary tract infection; and (4) no evidence of multiple stones or nephrocalcinosis in the computed tomography scan [7].

To date, since the literature on the subject is scarce, there are no definitive criteria for the surgical management of kidney stones from living donors. Allograft kidneys are solitary renal units, and management of urinary stones demands immediate intervention because these stones represent a potential threat due to the risks of obstruction, sepsis, and loss of graft function [8]. Herein, we report our surgical experience and long-term follow-up in a series of living kidney donors (LKD) with asymptomatic kidney stones, after ex vivo flexible ureterorenoscopy (f-URS) for stone removal before kidney transplantation.

## Materials and methods

A retrospective study including 1743 assessed LKDs at a single centre. During the pre-transplant evaluation, 18 (1%) LKDs were diagnosed with urolithiasis by computed tomography angiography (CTA). All donor candidates were asked about symptoms derived from the stones and a basic metabolic workup for renal stones was performed when urolithiasis was identified in the CTA. As a result, six (33%) patients went through with the kidney donation after ex vivo f-URS for stone removal. All were assessed regarding the risks related to the bench surgery and the complications due to renal stones and informed consent was signed.

### Surgical technique

**Fig. 1:**
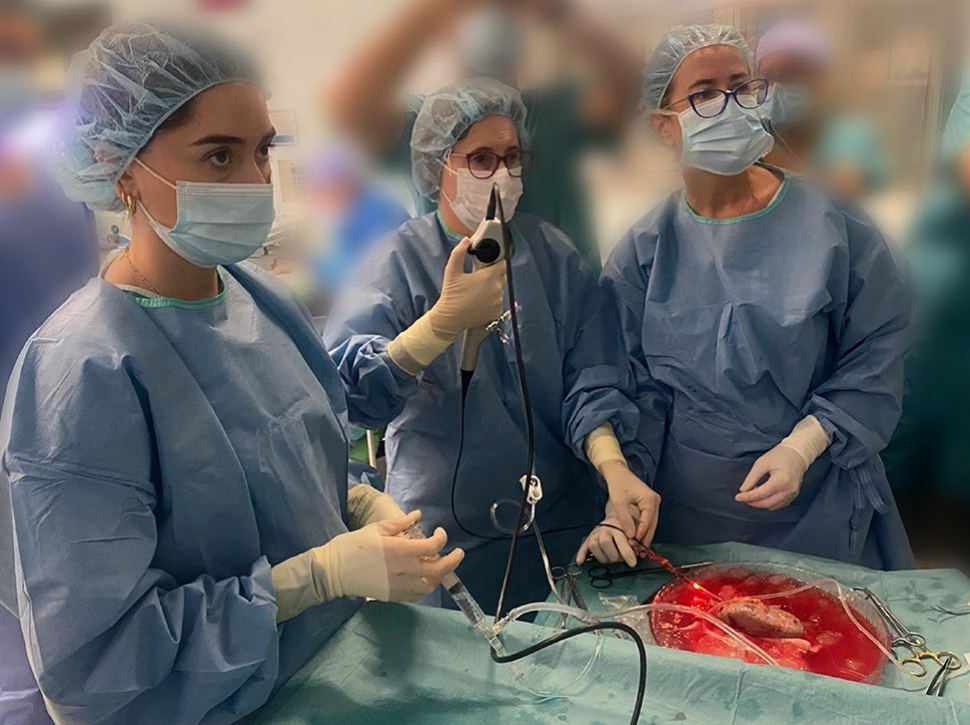
After the cold perfusion in the bench surgery, with the kidney graft immersed in ice slush the procedure starts with the introduction of a re-usable flexible ureteroscope with a constant irrigation at 40 cm H_2_O and a manual syringe allowing on-demand forced irrigation. The ureter and the kidney were fixed by the assistant and the introduction of the scope was gently assessed through the ureter reaching the renal pelvis

After adequate preparation and informed consent, a laparoscopic nephrectomy was performed as described previously [9]. After cold perfusion in the bench surgery, with the kidney graft still immersed in ice slush, the procedure began (Fig. 1). First, we spatulate the distal ureter. Each procedure begins with the introduction of a flexible re-usable fibreoptic ureteroscope -the URF-P7 (7.95 Fr, Olympus, USA) or the Flex—Xc (8.5 Fr, Karl Storz, Germany), with a constant 0.9% saline irrigation pressure (40 cm H_2_O) at ambient temperature and a manual syringe (Irri FloII, Olympus, USA), allowing on-demand forced irrigation when a better view is required. The ureter and the kidney are fixed by the assistant and the introduction of the scope is gently assessed through the ureter reaching the renal pelvis. All renal cavities are explored. When the stone is identified, we remove it using a basket (1.5 Fh, ZeroTip, BostonScientific, USA). In all cases, the stone is sent to the laboratory for FTIR spectroscopy and morphological analysis. All interventions are carried out by an experienced endourologist (PL). If this technique fails, pyelolithotomy is performed.

Once the kidney is completely stone-free, the graft is transplanted as described, by an open or Robotic-assisted (RAKT) technique [10]. Intravesical ureteroneocystostomy (Politano-Leadbetter) or extravesical ureteroneocystostomy (Lich-Gregoire) for an open or RAKT approach were used, respectively. A double J (6 Fr, 14 cm, Silicone, Coloplast) is placed after surgery and removed one month after the renal transplant.

### Data collection

Data were collected during surgery, including stone volume, Hounsfield units (HU), renal stone placement, and bench surgery time. Postoperative complications were evaluated according to the Clavien-Dindo classification. We also assessed graft follow-up by creatine controls and urinary stone recurrence in both donors and recipients.

## Results

A total of six living kidney transplants were analysed. Four (67%) LKDs and three recipients were female. Four donors (67%) were blood-related to the recipient. The median (IQR) age was 57.5 (44–62) and 51.5 (34–66) years for donors and recipients, respectively. All LKDs were previously asymptomatic, and the diagnosis was made incidentally by CTA during kidney transplant evaluation. Two donors had a history of urolithiasis, diagnosed, and surgically treated 20 years before. A single renal stone was identified in all. The median (IQR) size was 6 (4–13) mm. The median (IQR) stone density was 715 (189–1700) HU. Four lithiasis (67%) were in the lower calyx (Table 1). Urinary culture and metabolic evaluation were negative in all cases.

**Table 1:** Demographics of donor population and stone characteristics

S. no.	Donors–recipient’s relationship	Age (years)	BMI (kg/m^2^)	Comorbidities	Stone size (mm)	Stone density (HU)	Stone placement	Stone type	Stone related events post-donation
1	Wife	58	28.69	Pancreatitis	6	430	Lower left calyx	Whewellite 95% (Randall Plaque)	None
2	Wife	49	20.44	None	4	189	Lower left calyx	Whewellite 90% Weddellite 5% Carbapatite 5%	None
3	Sister	44	22.31	Urolithiasis (20 years)	8	880	Lower left calyx	Carbapatite 80% Whewellite 10% Proteins 10%	None
4	Mother	57	25.71	Dyslipidemia Hyperthyroidism	6	970	Middle right calyx	Weddellite 70% Whewellite 20%	None
5	Brother	62	27.78	HTA	13	550	Lower right calix	Whewellite 80% Uric acid 20%	None
6	Brother	60	27	Urolithiasis (20 years)	5.33	1700	Middle left calyx	Whewellite 95%	None

The fifth case was the most complex of the series, with a 13 mm stone in the lower right calyx. They were treated three months before with extracorporeal shock wave lithotripsy (ESWL), leaving small residual stones of 4 and 2 mm in the lower right calyx, which were all removed during bench f-URS. In one patient, we could not remove the 8 mm stone during bench f-URS due to no progression at the proximal ureter, consequently resorting to pyelolithotomy. Kidney stone analysis was performed in all cases, most of the calculi contained whewellite as the main component (66.7%) (Table 2).

**Table 2:** Demographics of recipient population and surgical outcomes

S. no.	Age (years)	Sex	BMI (kg/m^2^)	Comorbidities	Stone removal technique	Ischemia time		Delayed graft function	Immediate postoperative complications	Renal function (Cr, mg/dL)
						Cold (min)	Warm (min)			
1	63	Male	24.69	HTA, duodenal ulcer, diverticulitis	f- URS	46	2	No	Paralytic ileus	1.26
2	45	Male	24.8	None	f- URS	43	1.37	No	Arterial thrombosis (7 days after)	
3	45	Female	21.33	HTA, chronic anemia	f- URS + pyelolithotomy	41	3	No	Non	1.8
4	34	Female	18.8	HTA	f-URS	38	3	No	Wall hematoma	1.35
5	56	Male	26	Ischemic heart disease	SWL + f-URS	45	1.18	Yes	Non	1.52
6	66	Female	18	HTA, Diabetes Mellitus type I, sarcoidosis	f-URS	37	2.40	No	Non	1.19

HTA hypertension, SWL Shock wave lithotripsy

Median cold ischemia time was 41.6 (37–46) minutes with complete stone removal in all cases. Neither nonimmediate bleeding nor acute rejection was observed (Table 2). As immediate postoperative complications, one patient developed paralytic ileus managed with mobilisation and prokinetics. Another patient developed delayed graft function, being the two-stage case, with the longest cold ischemia time of the series. During follow-up, six years later, the patient still presented good kidney function, with 1.63 mg/dl creatinine levels and a stone-free status. Another patient presented good postoperative evolution, but seven days later presented signs of arterial thrombosis, confirmed by echo-Doppler, therefore, a transplantectomy was performed.

After a median (IQR) follow-up of 120 (2.75–359.2) months, the remaining grafts were functioning with a median (IQR) creatinine level of 1.42 (1.2–1.8) mg/dl, and none of the recipients or living donors presented urinary stone recurrence (Table 3).

**Table 3:** Management recommendation

Stone size (mm)	Management recommendation
< 4	Expectant management
4–6.5	URS + Graspers/Basket
7–10	URS + Pyelotomy
10–15	SWL + URS

## Discussion

Donor graft lithiasis is currently estimated to be approximately 0.64% [11] and was a relative contraindication for donation; however, nowadays it can be managed before, during, or after kidney transplantation [12]. In our study, kidney transplant recipients who received an allograft from living donors with kidney stones removed during bench f-URS had a very low recurrence rate and kidney graft survival was 83% after long-term follow-up, with one graft loss that was not directly related to stone management.

Due to the lack of literature, stone management in transplanted kidneys is a matter of debate. Current options are observation, ESWL, endourologic interventions, or percutaneous nephrolithotomy and open surgery. Conservative management is recommended for stones under 4 mm, however, 70% of expected spontaneous stone passage failed in a prior series [13, 14]. In addition, urolithiasis in renal transplant recipients is often asymptomatic due to denervation of the transplanted graft [14–16]. Clinical findings of urolithiasis in renal recipients include unexplained fever, increased creatinine levels, decreased urine output, and haematuria. Even with a longer cold-ischemia time, we press to treat the stone before transplantation to avoid stone-related events. Moreover, several factors have been considered to predispose graft lithiasis progression, such as urinary stasis, reflux, recurrent urinary tract infection, renal tubular acidosis, pH changes, supersaturated urine, etc., [5, 17]. Therefore, we consider lithiasis treatment before kidney transplantation to be essential.

Most series report outcomes and treatment of stone formation after kidney transplantation [6, 13], few series report their results after living donor-gifted lithiasis. The largest series was published by Jan et al., with 57 donor-recipient pairs [18]. The kidney with the stone was donated to the recipient and after a median follow-up of 3.5 years, 15.79% lost the allograft and 5.26% presented stone recurrence with no impact on posttransplant care and graft function. Pushkar et al., presented a series of 14 LKDs with stone sizes between 4 and 10 mm. According to stone size, only three patients were treated with f-URS during bench surgery, however, pyelolithotomy was required for larger stones [19]. Similar findings were produced in our series, the 8 mm stone could not be extracted via the distal ureter, hence pyelolithotomy had to be performed.

Urinary stones of between 10 and 15 mm in kidney grafts is a greater challenge. ESWL and endourology (percutaneous nephrolithotomy and URS) are grade B recommendations of the European Association of Urology (EAU) [20]. This is why in our series, the patient with a 13 mm stone was managed by a two-stage treatment.

A basic metabolic stone screening is also recommended by the EAU for all patients presenting kidney stones, which includes urine analysis, measurement of serum calcium, uric acid, and creatinine, and analysis of the stone [19]. None of the LKDs presented metabolic disruption in this evaluation and the kidney stone analysis revealed whewellite as the main component in most stones. Similar findings were described by Tielius et al., where 70–85% were calcium stones, 3–15% infective stones, and 2–18% uric acid stones [21].

On the other hand, donors without proven risk of recurrent stone formation may be suitable for donation if the stone size is under 15 mm and the kidney is anatomically suitable for transplantation [3]. In fact, in our series, none of the donors presented stone recurrence in the other kidney during follow-up. Our study reveals that asymptomatic kidney stone formers may not share the same burden of co-morbidities as has been described in symptomatic stone formers. Along that line, Burgher et al., performed a retrospective review of 300 male patients with asymptomatic renal calculi to evaluate the risk of progression requiring intervention [22]. At presentation, the mean cumulative stone diameter was 10.8 mm. After a mean follow-up of 3.2 years, 77% of patients experienced disease progression with 26% requiring surgical intervention, finding a positive association between stone size and progression. When stratified by stone size, patients with isolated stones of 4 mm or larger at presentation were 26% more likely to need an active treatment than patients with a smaller solitary calculus. The risk of stone recurrence and subsequent morbidity in renal patients with a solitary kidney is low but not insignificant, so a strict follow-up is mandatory.

We are aware that this is a retrospective study with a relatively small number of cases. Large prospective studies are difficult to perform because of the low incidence of allograft renal calculi in the kidney donor population. Despite this limitation, we have demonstrated the efficiency and safety of minimally invasive procedures in the treatment of incidental renal lithiasis in LKDs.

## Conclusions

Living donor grafted lithiasis is a quite rare event. Donors with asymptomatic incidental renal stones are generally accepted for donation. Therapeutic arsenal recommendation is based on the size of the stone, ex vivo f-URS with minimal morbidity can be performed without potential hazards to graft outcome. Adequate counselling and close follow-up are essential for both donors and recipients. Long-term studies with more patients are needed to establish the best therapeutic option for this specific category of patients and donors.

## Data Availability

Not applicable.
